# Supplementary material for: Trait-based meta-analysis of microbial guilds in the iron redox cycle
Source: mSystems. 2026 Jan 26;11(2):e01488-25. doi: 10.1128/msystems.01488-25 (PMC12911419; doi:10.1128/msystems.01488-25)
Supplement: Supplemental Material — Extended legend for Fig. 4, supplemental figures, and supplemental tables captions. [file msystems.01488-25-s0002.docx]

**Trait-Based Meta-Analysis of Microbial Guilds in the Iron Redox Cycle**

Díaz-González Fernando^1,2^, Rojas-Villalobos Camila^1,3^, Issotta Francisco^1,4^, Reyes-Impellizzeri Sofia ^1,2^, Hedrich Sabrina ^5^, D. Barrie Johnson^6,7,8^, Temporetti Pedro^9^*, Quatrini Raquel^1,10^*.

1 Centro Científico y Tecnológico de Excelencia Ciencia & Vida, Santiago, Chile.

2 Programa de Doctorado en Biotecnología y Bioemprendimiento, Facultad de Medicina, Universidad San Sebastián, Santiago, Chile

3 Programa de Doctorado en Biología Computacional, Facultad de Ingeniería, Arquitectura y Diseño, Universidad San Sebastián, Santiago, Chile

4 Departamento Genética Molecular y Microbiología, Facultad de Ciencias Biológicas, Pontificia Universidad Católica, Santiago, Chile

5 Institute of Biosciences, Technische Universität Bergakademie Freiberg, Germany.

6 School of Biological Sciences, Bangor University, Bangor LL57 2UW, United Kingdom

7 Faculty of Health and Life Sciences, Coventry University, Coventry, United Kingdom

8 Natural History Museum, London, United Kingdom

9 Instituto de Investigaciones en Biodiversidad y Medioambiente (INIBIOMA), Centro Regional Universitario Bariloche-UNComahue, CCT-Patagonia Norte, CONICET, San Carlos de Bariloche, Argentina.

10 Facultad de Ciencias, Universidad San Sebastián, Santiago, Chile

* Co-correspondence

Raquel Quatrini, rquatrini@cienciavida.org; raquel.quatrini@uss.cl

Pedro Temporetti, [temporettipf@comahue-conicet.gob.ar](mailto:temporettipf@comahue-conicet.gob.ar)

**ADDITIONAL FILES**

The following material is available online.

**Extended Figure Legend for Figure 4**

**Fig. 4. Correlative patterns of phenotypic and genomic traits and trait-based clustering across MIRC guilds. A**) Correlogram of monotonic (Spearman’s ρ) and distance-based (Mantel’s ρ) correlations among phenotypic traits (pH, temperature, Fe concentration) and genomic traits linked to Fe cycling inferred with FeGenie. Correlation strength levels were as follows: weak |0.1| to |0.3|, moderate |0.3| to |0.5| and strong |0.5| to |1.0|. Mantel tests indicated that pH optima correlated moderately with genomic trait dissimilarity in OXI (ρ = 0.31–0.32) and O/R guilds (ρ = 0.30), but more weakly in RED (ρ = 0.19–0.21). Temperature optima showed weak correlations with OXI and O/R guilds (ρ = 0.18–0.20), while Fe concentration optima correlated weakly with RED and O/R (ρ = 0.15). **B**) Heatmap-based hierarchical clustering of 208 taxa (a representative subset of the 387 spp. with available data) using 15 variables, including optimal environmental conditions (phenotypic variables; pH, temperature, Fe concentration) and 12 Fe-related genomic traits. The phenotypic variables were standardized and the genomic variables were normalized between 0 and 1. Then, a matrix with Euclidean distance was calculated and hierarchically grouped with the McQuitty method. Six major clusters were resolved, reflecting distinct trait syndromes: i) Cluster 1 (n = 101): Metabolically versatile mesophiles spanning wide pH ranges (4.75–9.25), with broad capacities for Fe oxidation/reduction, siderophore transport, and regulation — representing generalist guilds; ii) Cluster 2 (n = 8): Extreme acidophiles (pH 1.1–2.5) consistently encoding Fe oxidation genes, adapted to highly acidic niches; iii) Cluster 3 (n = 36): Thermophiles (50–72.5 °C) in neutral to slightly alkaline habitats, enriched in Fe transport, siderophore pathways, and storage functions but lacking Fe oxidation genes, reflecting Fe acquisition–dominated strategies; iv) Cluster 4 (n = 22): Mesophilic to moderately thermophilic acidophiles (pH 1.1–4.25) with strong enrichment in Fe oxidation and siderophore transport genes, specialized for oxidative metabolism at low pH; v) Cluster 5 (n = 22): Moderate to hyperthermophiles (59–100 °C), split by pH. Acidophilic members carry Fe oxidation genes, while circumneutral taxa lack oxidation capacity but encode Fe reduction and acquisition pathways — demonstrating ecological divergence driven by pH; vi) Cluster 6 (n = 8): Mesophiles with variable pH optima enriched in siderophore transport, reflecting iron-scavenging strategies across heterogeneous environments.

**Figure S1 (Figure_S1.pdf). Morphological and taxonomic description of MIRC guilds.** **A**) Frequency in balloon visualization of morphological adaptations of MIRC guilds. Differentiated by Gram stain response (upper panel, Positive, crystal violet, blue-magenta color; Negative, safranin, pink-red color) and Spore-forming capability (lower panel, Forming, malachite green, cyan shadow color; Non-forming, safranin, pink-red color). The redox guilds were colored as: Fe(III)-reducing (**RED = cyan-blue**), Fe(II)-oxidizing (**OXI = pink-red**) and Fe-oxide/reducers (**O/R = yellow**). **B**) Frequency of sankey flows visually connects major taxonomic ranks, from Domain to Class, in the guild. The width of the flow paths corresponds to the frequency of each community.

**Table S1 (Table_S1.xlsx). Microbial Iron Redox Cycle representatives included in the metanalysis.** Species-level assignments of *387 MIRC spp. (documented across 314 publications)* to first-order guilds: Fe(III)-reducers (RED), Fe(II)-oxidizers (OXI), and dual-capacity Fe-oxidizers/reducers (O/R). Each entry includes the guild assignment, NCBI Taxonomy ID, 16S rRNA accession number used for phylogenetic inference, genome accession number used for trait annotation, and the primary reference describing the isolate or genome sequence.

**Table S2** **(Table_S2.xlsx).**  **Habitat distribution and source material of first-order microbial iron redox cycle (MIRC) guilds.** Microbial taxa categorized as Fe(III)-reducers (RED), Fe(II)-oxidizers (OXI), and dual-capacity Fe-oxidizers/reducers (O/R) were enumerated according to (A) their reported environmental origin and (B) the type of source material from which they were isolated or described, based on literature and/or genomic metadata. Habitats span aquatic, sedimentary, soil, hydrothermal, acid mine drainage, and other specialized ecosystems, with the “Most frequent habitat” column highlighting predominant environments for each guild. Source material was classified into particles (minerals, mud, organic matter, sludge, soil), fluids (water column or porewater), and biofilms (surface-associated mats). The table S2b reports both cumulative frequencies and relative percentages for RED, OXI, and O/R guilds, with the “Source material usage (%)” row indicating the proportion of taxa for which metadata on source type were available (n = 8 categories scored).

**Table S3 (Table_S3.xlsx). Trait syndromes representing the most recurrent lifestyle configurations within first-order MIRC guilds.** Microbial taxa categorized as Fe(III)-reducers (RED), Fe(II)-oxidizers (OXI), and dual-capacity Fe-oxidizers/reducers (O/R) were evaluated for co-occurring sets of ecophysiological traits, interpreted here as trait syndromes. These syndromes reflect guild-specific adaptations to distinct environmental conditions and are reported as the most frequent combinations of metabolic type, electron donor preference, carbon assimilation strategy, oxygen tolerance, pH preference, temperature range, and salinity tolerance.
