# Supplementary material for: Trait-based meta-analysis of microbial guilds in the iron redox cycle
Source: mSystems. 2026 Jan 26;11(2):e01488-25. doi: 10.1128/msystems.01488-25 (PMC12911419; doi:10.1128/msystems.01488-25)
Supplement: Figure S1 — Morphological and taxonomic description of MIRC guilds. [file msystems.01488-25-s0001.pdf]

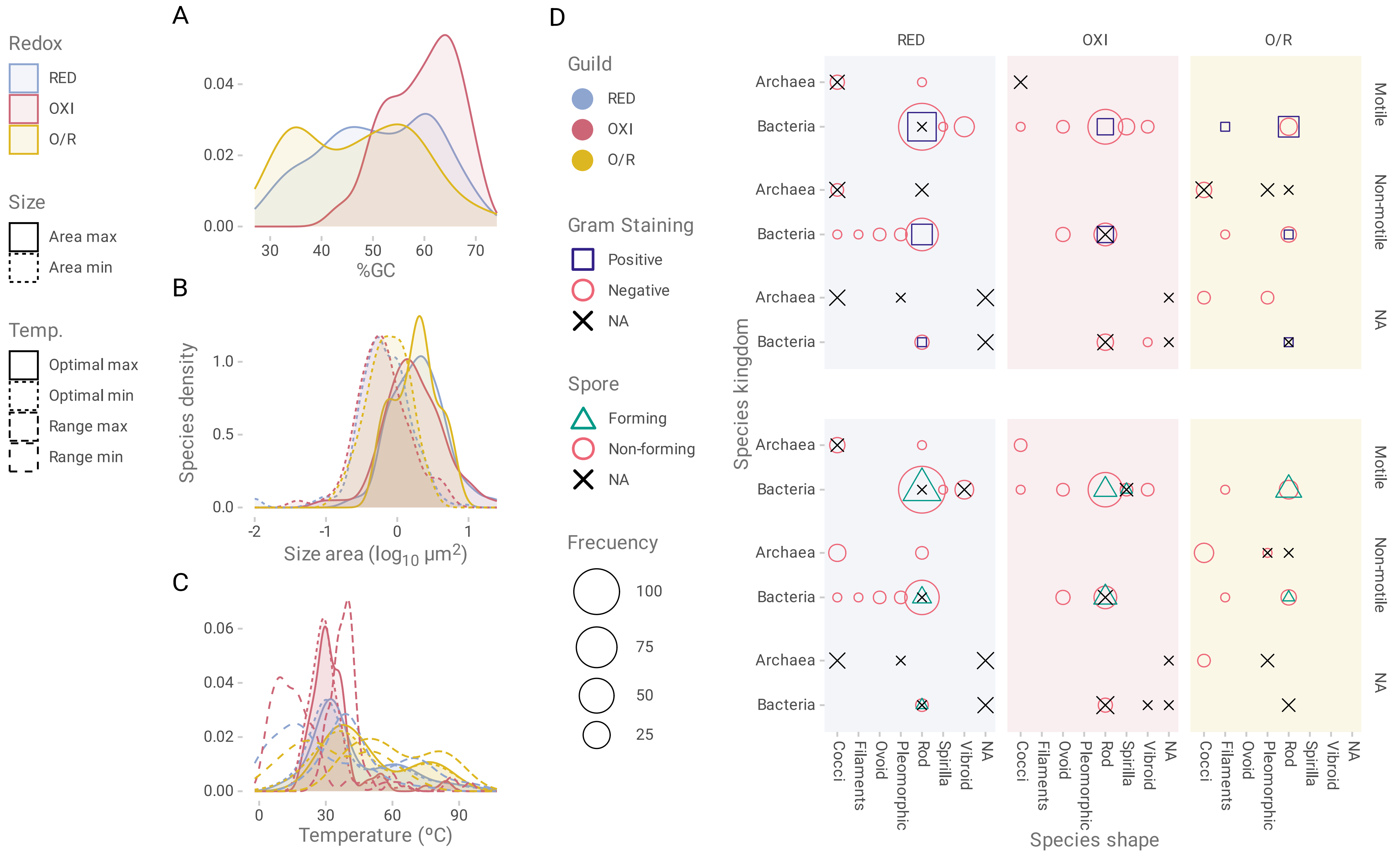

A
B
C
D
Redox
RED
OXI
O/R
Size
Area max
Area min
Temp.
Optimal max
Optimal min
Range max
Range min
Species density
%GC
Size area ($\log_{10}$ μm^2)
Temperature (°C)
Guild
RED
OXI
O/R
Gram Staining
Positive
Negative
NA
Spore
Forming
Non-forming
NA
Frecuency
100
75
50
25
Species kingdom
Archaea
Bacteria
Motile
Non-motile
NA
Species shape
Cocci
Filaments
Ovoid
Pleomorphic
Rod
Spirilla
Vibroid
NA
